# Evaluating the Limitations of One‐Dimensional High‐Temperature Gas Chromatography ‐ FID for Wax Solution Analysis: A Systematic Study

**DOI:** 10.1002/jssc.70382

**Published:** 2026-03-04

**Authors:** Fernando B. Okasaki, Ivanei F. Pinheiro, Letícia Bizarre, Vanessa C. B. Guersoni

**Affiliations:** ^1^ Faculdade de Engenharia Mecânica Universidade Estadual de Campinas (UNICAMP) Campinas São Paulo Brazil; ^2^ Centro de Estudos de Energia e Petróleo (CEPETRO) Universidade Estadual de Campinas (UNICAMP) Campinas São Paulo Brazil

**Keywords:** high‐temperature gas chromatography with flame ionization detection (HTGC–FID), paraffin wax characterization, sample preparation limitations, wax deposition

## Abstract

Wax deposition in petroleum systems is intrinsically connected with paraffin wax composition, yet their quantitative characterization by one‐dimensional high‐temperature gas chromatography with flame ionization detection (HTGC–FID), as prescribed by ASTM D5442, remains challenging. In this work, we systematically quantify the main sources of error affecting HTGC–FID analysis of paraffinic systems and benchmark the chromatographic results against differential scanning calorimetry (DSC). The results demonstrate that solvent‐based sample preparation leads to nonhomogeneous solutions at the colloidal scale due to paraffin aggregation, introducing significant sampling errors that intensify with increasing molecular weight, whereas an additional error source arises from incomplete volatilization of heavy paraffins in HTGC. A trade‐off between loss of detectability at high dilution and aggregation effects at high concentration was observed, impacting quantitative analysis. The absence of reliable retention time patterns for nonlinear paraffins highlights the intrinsic limitations of one‐dimensional HTGC–FID for their structural assignment, emphasizing the need for more advanced chromatographic approaches for comprehensive wax characterization.

## 1 Introduction

Wax deposition is one of the main flow assurance challenges faced by the oil and gas industry. In a pessimistic scenario, production pipelines become fully clogged with wax and mechanical removal has to be dispatched, leading to significant production and financial losses. There is currently a renewed focus on understanding the wax deposition phenomenon from a fundamental mechanistic perspective. This understanding would enable the development of accurate predictive models to efficiently prevent or mitigate the issue [1, 2, 3].

The process of wax deposition is inherently complex and influenced by two main groups of variables. The first group is related to environmental factors, including the flow regime and the temperature gradient to which the system is subjected. The second group involves the intrinsic properties of the waxy fluid, such as the wax appearance temperature (WAT), its rheological behavior, and the fluid composition [1, 2].

With respect to the fluid composition, attention is needed when characterizing the paraffin waxes within the fluid. Paraffins are hydrocarbons with a carbon number (C#) higher than 18, classified within the “saturates” group in the saturates, aromatics, resins, and asphaltenes (SARA) analysis [2]. The C# distribution profile of a paraffinic fluid defines the crystalline behavior of the system. If most compounds are linear paraffins (*n*‐paraffins) with a C# in the range of 18–40, the system is referred to as macrocrystalline. In such systems, paraffins can efficiently pack and tend to form elongated plate‐like crystals, increasing the viscosity of the system. Conversely, the presence of nonlinear paraffins, such as isoparaffins or naphthenes, disrupts the efficient packing of a macrocrystalline system. This disruption leads to the formation of a microcrystalline system, where paraffins form smaller spherical crystals, resulting in substantially less viscous systems compared to macrocrystalline systems [4]. Another relevant source for microcrystallinity is the presence of elevated polydispersity regarding the C# and, consequently, the molecular weight. In systems containing both light paraffins and heavy paraffins (C# > 40), the significant differences in chain length hinder efficient crystal packing, promoting the formation of microcrystalline structures [5].

Hydrocarbons are typically volatile compounds; however, paraffins comprise relatively large molecules with high molecular weights and therefore exhibit moderate to low volatility. This characteristic necessitates the use of high‐temperature gas chromatography (HTGC) to achieve adequate analytical performance in the fluid characterization. Although numerous studies in the literature addressing crude oils and wax deposition employ HTGC for paraffin characterization, only a small fraction of these works place primary emphasis on detailed chemical characterization of the fluid. In most cases, HTGC is used as a supporting technique, and its results are presented with limited discussion. Significant advances in equipment, tools, and techniques have substantially improved the efficiency of chromatographic analysis in petroleum systems. Comprehensive two‐dimensional gas chromatography (GC×GC) offers greater chromatographic space, enabling the detection and quantification of numerous compounds that would otherwise co‐elute in one‐dimensional GC [6, 7]. Coupling GC×GC or HTGC×GC with mass spectrometry enables a deeper analysis of the diverse molecular families within petroleum hydrocarbons, whereas the use of soft ionization techniques (e.g., electrospray ionization) enables the characterization and quantification of polar molecules containing heteroatoms, which is a relevant information for the prevention of equipment corrosion and emulsion formation [6, 8, 9].

Examples of studies in the literature that place chromatographic characterization of crude oils as a central objective are cited below. These works account the full range of molecular structures and molecular weights of paraffin waxes [10].

The work of França et al. employed comprehensive two‐dimensional gas chromatography (HTGC×GC‐flame ionization detection [FID] and HTGC×GC–MS) to fully characterize Brazilian whole crude oils [11]. The authors successfully quantified diverse classes of nonlinear paraffins. It is noteworthy that, although nonlinear compounds are present at much lower individual concentrations than *n*‐paraffins, their combined concentration can exceed the total concentration of *n*‐paraffins. Therefore, neglecting these compounds may lead to significant errors in estimating the total paraffin content of a sample. A subsequent study by the same authors further advanced crude oil characterization by quantifying *n*‐paraffins, isoparaffins, monocyclic and polycyclic naphthenes, mono‐ and polyaromatic species, and polar organic molecules [8].

Another example of research focused on the application of HTGC for paraffin wax characterization is the work developed by Paso et al. In this work, the authors investigated the aging behavior of wax deposits, a key process associated to the temporal evolution of its composition and physicochemical properties. HTGC was employed to inspect the effect of varying thermal conditions and the presence of isoparaffins on the determination of the critical carbon number (CCN), a central parameter to comprehend the aging behavior [12, 13].

It is also worth noting that several instrumental advances have been implemented in the chromatographic application of the SARA analysis for crude oils, which has traditionally relied heavily on bench procedures involving extensive use of solvents and laboratory materials. In this context, thin‐layer chromatography coupled with FID (TLC‐FID) has emerged as a valuable alternative, offering a faster, less resource intensive, and comparably efficient analytical methodology [14, 15, 16].

From a practical perspective, a large portion of paraffin wax characterization is still performed using one‐dimensional HTGC, as this is the methodology prescribed by ASTM D5442 [17]. In this context, a common oversight in the literature is the neglect of nonlinear paraffins and heavy paraffins with carbon numbers exceeding the macrocrystalline range (C# > 40). This simplification often results in treating the paraffins as exclusively *n*‐paraffins with a C# between 18 and 40. Such an approach constitutes a significant limitation, as the detailed paraffin composition directly influences the crystalline behavior of the system and, consequently, its rheological and thermal properties.

When using one‐dimensional HTGC two main sources of error must be considered. The first arises from instrumental limitations in achieving sufficiently high temperatures for the quantitative characterization of paraffins, particularly nonvolatile heavy paraffins. The second is associated with sample preparation, as conventional methodologies, including that prescribed by ASTM D5442, rely on solvent‐based paraffin solutions prior to injection. Ashbaugh et al. demonstrated by small‐angle neutron scattering (SANS) measurements that, even at temperatures above the wax dissolution temperature (WDT), paraffin molecules can remain aggregated in solution. Thus, although no longer in a solid state, they cannot be considered as fully solubilized [18].

Several studies have addressed the comparison of different paraffin extraction methodologies or sample pretreatments procedures for evaluating the chemical composition of paraffin waxes in crude oil samples. The sample matrix of crude oil is markedly complex and distinct extraction methodologies can represent distinct production processes that the crude oil undergoes [19, 20, 21, 22]. Oliveira et al. proposed an alternative sample preparation strategy based on direct matrix introduction (DMI) in contrast to conventional solvent‐based methodologies, significantly improving analytical performance for samples containing heavy paraffins [23].

In this work, we provide a quantitative assessment of the limitations of one‐dimensional HTGC in the quantification of paraffin waxes. An experimental setup similar to that prescribed by ASTM D5442 was employed. The chromatographic results were compared with those obtained using a well‐established differential scanning calorimetry (DSC) method, which is commonly used to determine paraffin content in crude oils. On the basis of this comparative analysis, we propose interpretations regarding the relative contribution of the main factors that can drive the analytical errors in HTGC. Given the substantial influence of the paraffinic composition on the physicochemical properties of waxy systems, we believe that this work will assist the O&G community in selecting appropriate experimental setups for paraffin characterization, understanding the inherent limitations of the methodology and more accurately interpreting results obtained from one‐dimensional HTGC–FID analyses.

## 2 Methodology/Application

### 2.1 Materials and Sample Preparation

Stock solutions were prepared with the following solutes: n‐hexadecane (Sigma‐Aldrich ≥ 95.0%), *n*‐eicosane (Sigma‐Aldrich ≥ 99.5%), *n*‐triacontane (Supelco ≥ 98.5%), *n*‐tetracontane (Sigma‐Aldrich ≥ 95.0%), *n*‐pentacontane (Sigma‐Aldrich ≥ 98.0%), iso‐hexadecane (2,2,4,4,6,8,8‐heptamethylnonane) (Sigma‐Aldrich ≥ 95.0%), decylcyclohexane (TCI ≥ 98.0%), and decylbenzene (TCI ≥ 95.0%). Macrocrystalline wax (melting point 59.0°C–62.8°C) and microcrystalline wax (melting point 76.7°C–87.8°C) were obtained from Solven, commercially named WAX 140 and WAX 170, respectively. Cyclohexane (AppliChem ITW reagents, HPLC grade ≥ 99.9%) was used as solvent for all samples.

Stock solutions were prepared gravimetrically using an analytical balance, weighing the required amounts of each solute and cyclohexane to achieve the desired weight percentage. Each stock solution was used to prepare diluted solutions, which underwent HTGC analysis. Before the preparation of the diluted samples, all stock samples were maintained in an oven at 60°C for 2 h to enhance solubilization and homogeneity within the stock solutions. The dilutions were also prepared gravimetrically immediately after the removal of the stock solution from the oven, by weighing the desired amounts of stock solution. Then, cyclohexane was added to the desired final concentration.

### 2.2 High‐Temperature Gas Chromatography

All samples were analyzed using an Agilent 8890 Gas Chromatograph equipped with a DB‐5 HT column (30 m × 320 𝜇m × 0.1 𝜇m) obtained from Agilent. Helium was used as carrier gas with a flow rate of 4 mL min^−1^. A FID detector was employed using hydrogen (H_2_) as fuel with a flow rate of 30 mL min^−1^, synthetic air was used as oxidizer with a flow rate of 400 mL min^−1^, having approximately 10:1 oxidizer‐fuel flow rate ratio. Nitrogen (N_2_) was used as makeup gas with a flow rate of 25 mL min^−1^. An Automatic Liquid Sampler (ALS) equipped with a 10 𝜇L syringe was used to transfer the samples to the chromatograph. A split/splitless injector was used in the splitless mode with septum purge flow of 3 mL min^−1^ and purge flow to split vent of 75 mL min^−1^ at 1 min. The experiments from Sections 3.1 to 3.2 were performed in triplicate. The experiments from Section 3.3 were performed in duplicate.

The experimental parameters were optimized to achieve the highest possible sample representativity. Two evaluation criteria were adopted: For repeated injections of the same sample, the optimal experimental condition was defined as the one that produced the largest peak areas and enabled detection over the widest C# range. The data obtained in this parameter optimization are presented in the Supporting Information. The optimized experimental conditions employed in the HTGC experiments are summarized in Table 1.

**TABLE 1:** Experimental parameters used in high‐temperature gas chromatography (HTGC).

Parameters	Values
Injection volume/𝜇L	1
Temperature range/°C	90–380
Inlet temperature/°C	380
FID temperature/°C	390
Temperature ramp/°C min^−1^	8

Abbreviation: FID, flame ionization detection.

### 2.3 Quantification Methodology—HTGC

Calibration curves were used to quantify the paraffin compounds in the studied samples. The calibration curves were constructed using an ASTM D5442 quantitative linearity standard from Sigma‐Aldrich, which contains linear paraffin compounds with the following C#: 12, 14, 16, 18, 20, 22, 24, 26, 28, 30, 32, 36, 40, 44, 50, and 60, each at a concentration of 0.01 wt.% in cyclohexane.

The calibration curves were designed to cover the concentration range containing the diluted samples. Each curve was constructed with at least seven concentration points of paraffin weight percentage (4 × 10−5, 8 × 10−5, 2 × 10−4, 4 × 10−4, 8 × 10−4, 1 × 10−3, and 8 × 10−3). For C44, C50, and C60, the two most diluted points were excluded as they fell outside the linear range. All curves were constructed in triplicate.

The constructed calibration curves were fitted using linear regression, achieving Pearson correlation coefficients (*R* ^2^) of at least 0.995. To ensure consistency with the physical meaning of the data, the intercept of the linear fit was set to zero. To cover the full range of C# in the samples, the experimental slopes derived from the calibration curves were adjusted with a polynomial function using C# as the independent variable. The resulting adjusted curve and corresponding equation are presented in Figure S5. The polynomial fit achieved a Pearson correlation coefficient (*R* ^2^) of 0.998.

### 2.4 Differential Scanning Calorimetry

The DSC experiments were conducted in a Q2000 calorimeter from TA Instruments. The samples were loaded into an aluminum pan, which was then sealed and inserted into the DSC instrument. The DSC run started at 70°C and was cooled to −10°C at the rate of 1°C min^−1^, tracking all the thermal events that occurred in this temperature range. Before the experiment, the calorimeter was calibrated using an indium standard. Nitrogen was used as purge gas in the calorimeter cell.

Data were treated using TA Universal Analysis software. The paraffin content was obtained through the integration of the crystallization peak, using methodologies presented in the literature [13, 24].

## 3 Results and Discussion

In this work, three variables relevant to the study of wax deposition are investigated: the molecular weight of the paraffinic compounds, the system crystallinity, and the molecular structure of the paraffinic compounds. The molecular weight distribution significantly influences the aging behavior of the deposit [13]. The system crystallinity affects several of its physicochemical properties [4, 5]. Finally, the molecular structure of the paraffinic compounds, which is often oversimplified, plays a critical role in determining the system crystallinity [8].

### 3.1 Effect of Paraffin Molecular Weight

**FIGURE 1:**
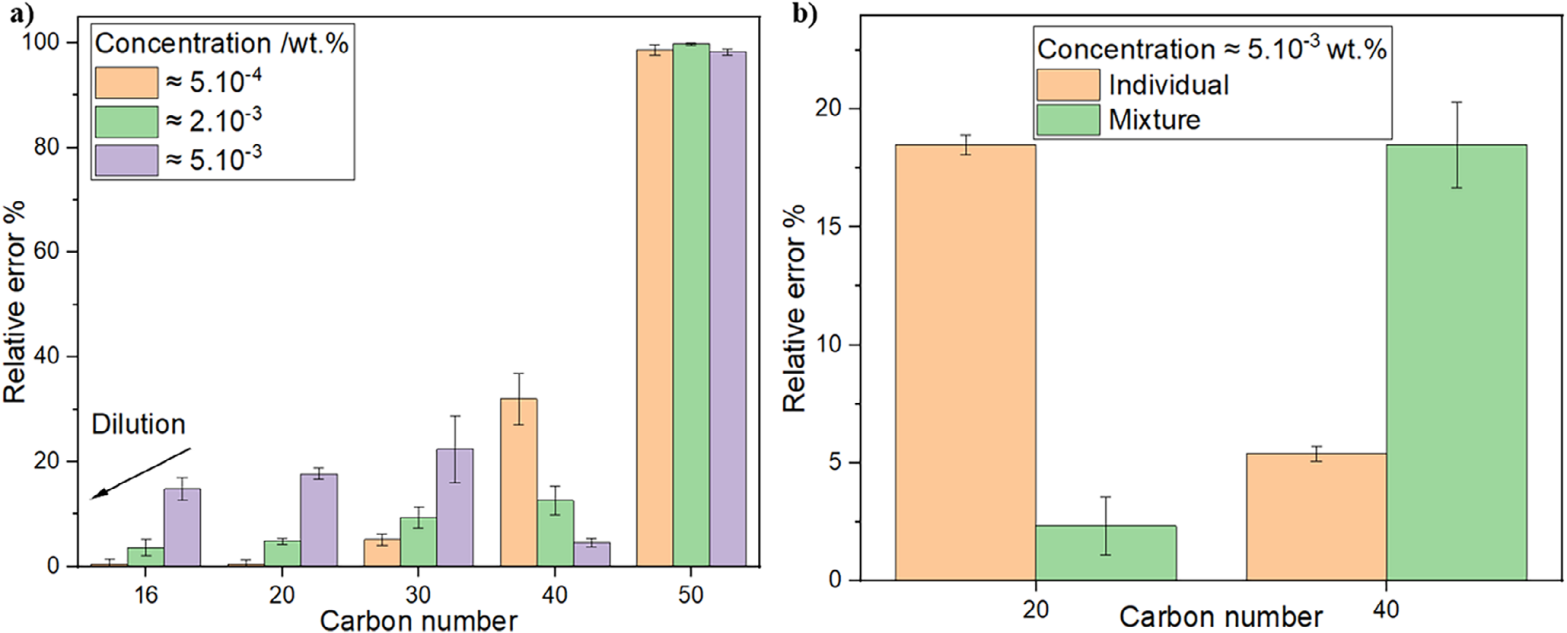
(a) Relative error in concentration obtained for each carbon number. The theoretical concentrations are showed in the inlet. (b) Comparison between the relative error in HTGC analysis of C20 and C40 solutions when individually solubilized or in a mixed solution.

The first variable examined is the molecular weight of the paraffins. For that, 1 wt.% stock solutions of linear hydrocarbons (C16, C20, C30, C40, and C50) were individually prepared. Each stock solution was subsequently diluted to concentrations of 5 × 10−4, 2 × 10−3, and 5 × 10−3 wt.%. The diluted samples were then analyzed by HTGC. Experimental concentrations were determined using the corresponding calibration curves. These values were compared with the theoretical concentrations of each diluted solution, and the relative error was calculated using Equation (1), where 𝐶exp is the experimental concentration and 𝐶theo is the theoretical concentration. The relative errors as a function of concentration and carbon number are presented in Figure 1a.

(1) $$\mathrm{Relative}\;\mathrm{error}\left(\%\right)=\left|\frac{C_{\exp}-C_{\mathrm{theo}}}{C_{\mathrm{theo}}}\right|\times 100$$

Several considerations can be drawn from the results presented in Figure 1a. The overall trend indicates that, as the C# increases, the relative error also increases. This behavior is likely attributable to the decreasing solubility of paraffins as the C# and consequently the molecular weight increase. The relative errors observed for C50 are particularly pronounced, as this compound was almost undetectable under the experimental conditions employed. This is especially relevant for the characterization of unknown samples, as the presence of heavy paraffins significantly affects the physicochemical behavior of waxy systems [25]. Indeed, alternative methodologies have been reported in the literature to quantify paraffins with C# above 50 using gravimetric approaches, explicitly acknowledging the limitations of HTGC in efficiently analyzing such high‐molecular‐weight species [26].

**FIGURE 2:**
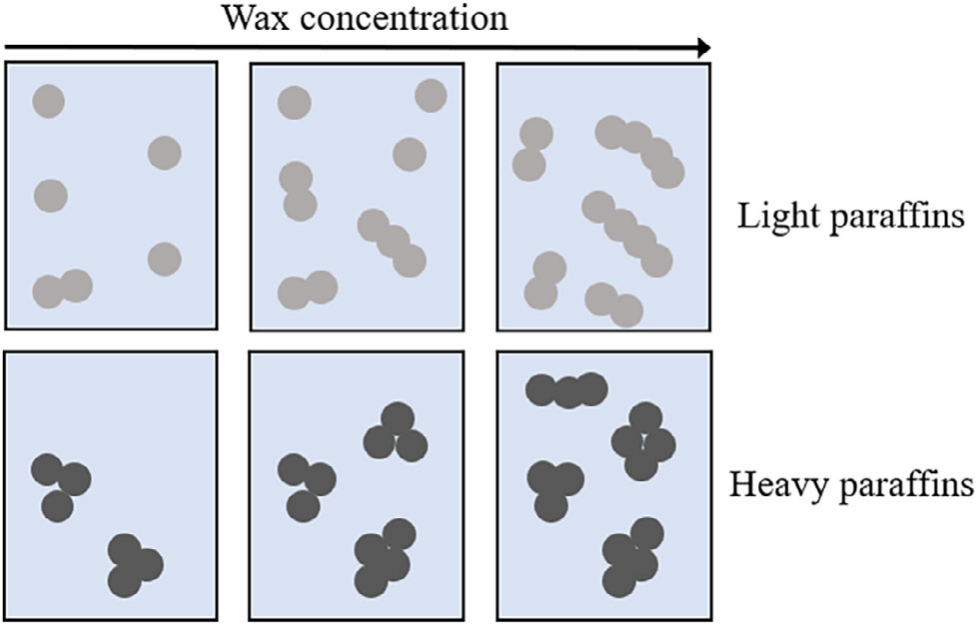
Schematic representation of the distinct aggregation behavior of light and heavy paraffins.

A noticeable change in behavior is observed between C30 and C40, which coincides with the C# range above which several studies in the literature discontinue chromatographic analysis for the characterization of waxy fluids. For C# below C30 (light paraffins), sample dilution leads to a reduction in relative error. Conversely, for C# values above C30 (heavy paraffins), increasing the sample concentration decreases the relative error. This contrasting behavior between light and heavy paraffins can be attributed to differences in solubility and sample homogeneity. For light paraffins, the extent of molecular aggregation in solution is comparatively low; thus, increasing concentration promotes aggregation, reduces sample homogeneity, and worsens sampling representativeness for GC analysis. In contrast, heavy paraffins exhibit a substantial degree of aggregation even under the most diluted conditions, which explains the persistently high relative errors observed for these samples. Increasing the concentration of heavy paraffin solutions does not significantly worsen aggregation; rather, it increases the number of aggregates present in the injected aliquot, which improves the statistical representativeness of sampling and, consequently, reduces the relative error. This phenomenon is schematically illustrated in Figure 2.

Turbidity measurements were performed in order to obtain additional experimental evidence supporting the aggregation hypothesis. The methodology and results of the turbidity measurements are presented in the Supporting Information. No significant differences in the turbidity of the samples were observed comparing the pure solvent and C16 or C40 solutions at 5 × 10−3 wt.%. This result suggests that the formed aggregates are on the nanometric scale, as previously detected through SANS [18]. Consequently, the aggregate sizes fall below the detection limit of light scattering techniques and no measurable turbidity is observed. Nevertheless, having macroscopic clear solutions does not guarantee homogeneity in the colloidal scale.

The works of Dirand et al. and Senra et al. studied the impact of polydispersity on the crystallization of *n*‐alkanes in solution. The authors observed that the presence of high molecular weight paraffins enhanced the solubility of low molecular weight paraffins [27, 28]. On the basis of these findings, we examined the relative error of the chromatographic analysis in a system containing both C20 and C40, the results are shown in Figure 1b. The results indicate that the presence of C40 significantly reduces the relative error of C20, even at high concentrations, as the improved solubility of C20 contributes to better homogenization of the system.

As also reported by Dirand and Senra, although the solubility of light paraffins is enhanced by the presence of heavy paraffins, the reciprocal effect does not occur. That is, the presence of light paraffins does not enhance the solubility of heavy paraffins, which is reflected in the increase of the relative error for C40 in the mixed system shown in Figure 1b, where the increase in the overall paraffin concentration likely promotes the aggregation of C40, leading to higher relative errors.

### 3.2 Effect of the System Crystallinity

**FIGURE 3:**
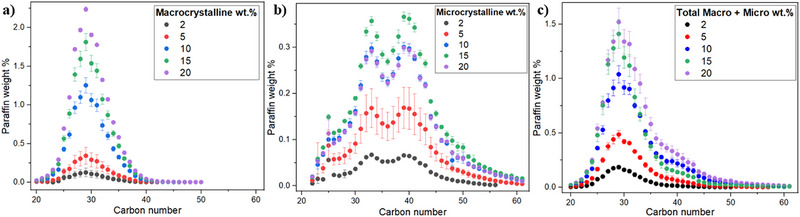
Determined concentration of each paraffinic compound in the systems: (a) macrocrystalline wax, (b) microcrystalline wax, and (c) 1:1 mixture of macrocrystalline and microcrystalline waxes. The concentrations of the stock solutions are shown in the inlet.

This section evaluates the performance of HTGC in the characterization of macrocrystalline and microcrystalline paraffin systems. The chromatograms for each system are given in Figure S6. All stock solutions were diluted to the concentration of ≈2 × 10−1 wt.% for the HTGC analysis. Figure 3 presents the HTGC results for the quantification of individual paraffinic compounds in the pure macrocrystalline and microcrystalline samples, as well as in the 1:1 mixed system.

The C# distribution follows an approximately normal profile for the macrocrystalline system (Figure 3a) and a bimodal profile for the microcrystalline system (Figure 3b), which is typical of paraffinic fluids [6]. For all three systems, the detectable C# interval becomes narrower when the most diluted stock solutions are used. This occurs because compounds located at the borders of the distribution are present at lower concentrations and, upon dilution, their concentrations fall below the HTGC detection limit. Although these species are individually present in small amounts, neglecting them can introduce significant errors in the estimation of the total paraffin content of the sample. A notable difference between the macrocrystalline and microcrystalline systems is observed in the order of magnitude of the determined paraffin mass fractions on the *y*‐axis. Despite both stock solutions having the same nominal concentration, the concentration of each individual paraffinic compound in the microcrystalline system is almost five times lower than in the macrocrystalline system. Because the average carbon number of the microcrystalline system is significantly higher, ineffective solubilization is more pronounced, leading to poor sample homogenization and increased relative error in HTGC due to irregular sampling. The extent of aggregation can be so severe that the paraffin mass determined for the 15 wt.% microcrystalline stock solution appears to be higher than that obtained for the 20 wt.% solution. Consequently, if the stock concentrations were unknown, the chromatographic analysis could misleadingly suggest that the 15 wt.% solution contains more paraffin than the 20 wt.% solution.

The mixed system (Figure 3c) contains the same total paraffin content as the pure macrocrystalline and microcrystalline systems, with the composition equally divided (1:1) between the two groups. The determined concentrations of each paraffinic compound exhibit magnitudes similar to those observed in the pure macrocrystalline system. This reinforces the interpretation that the presence of heavy paraffins, which are the main components of the microcrystalline sample, does not promote the aggregation of light paraffins; instead, it enhances their solubilization. When examining the data in Figure 3c, an intriguing question arises: Does the determined paraffin concentration in the mixed system at 10 wt.% correspond to the sum of concentrations observed in the pure macro or microcrystalline at 5 wt.% each?

**FIGURE 4:**
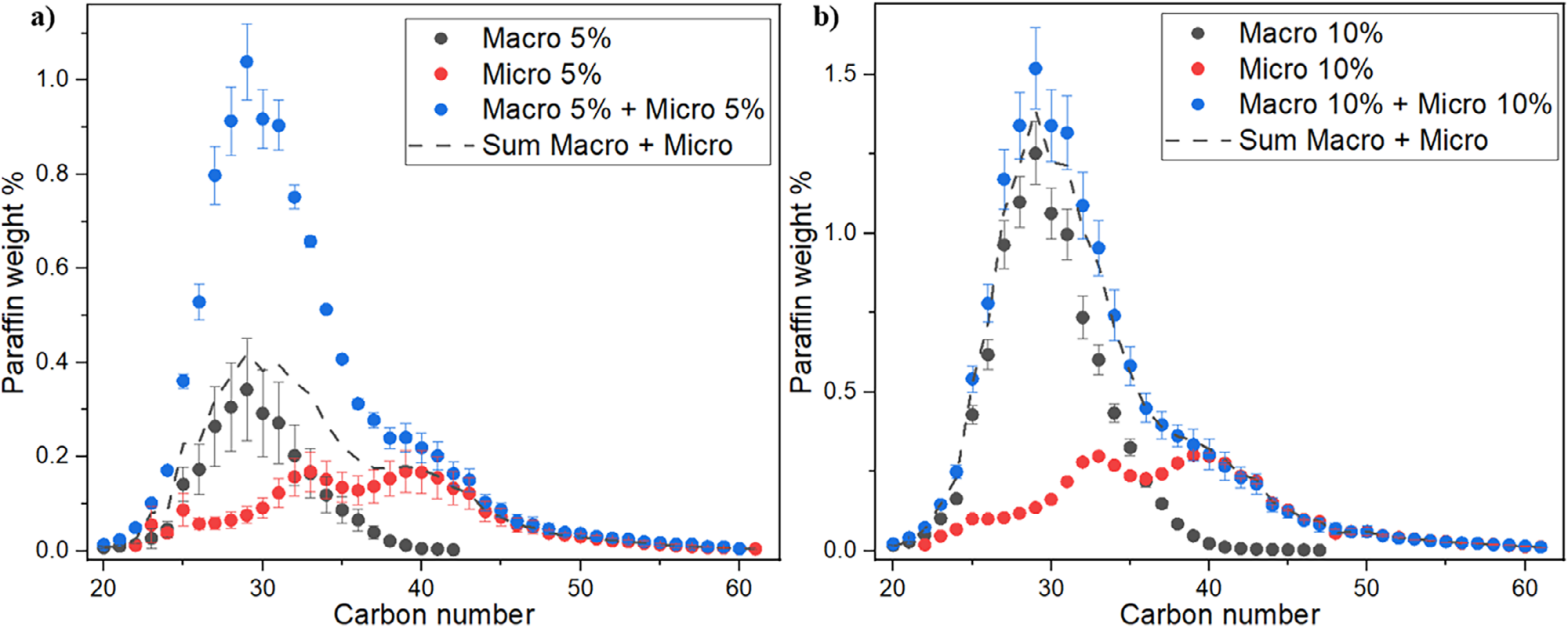
Comparison between the determined paraffin concentrations in (a) macro 5 wt.%, micro 5 wt.%, and mixed 10 wt.% and (b) macro 10 wt.%, micro 10 wt.%, and mixed 20 wt.%. The sum of the concentrations in the pure systems are shown in the dashed line.

The answer to this question is presented in Figure 4, which leads to two key observations. First, since the experimentally determined concentrations in the mixed sample exceed the sum of the corresponding pure samples, this provides further evidence that the presence of heavy paraffins, predominantly found in the microcrystalline paraffin, enhances the effective solubility of light paraffins, thereby improving the quality of the HTGC analysis. Second, the deviation between the sum of the concentrations of the pure samples and the concentration in the mixed sample, both experimentally determined, is more pronounced in the diluted scenario (Figure 4a) compared to the concentrated scenario (Figure 4b).

**FIGURE 5:**
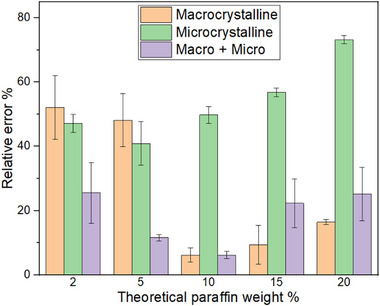
Calculated relative errors concerning the total paraffin content in pure macro or microcrystalline system or mixed system. The analysis was carried out by diluting all the stock solutions by a factor of 640.

To further investigate the relationship between sample concentration and the relative error in HTGC analysis, the relative errors associated with the total paraffin content in each pure and mixed system were calculated. The results are presented in Figure 5.

Analysis of Figure 5 shows that, for all three systems, the relative error increases at both extremes of concentration, under the most diluted and the most concentrated conditions, following an approximately parabolic trend. This behavior can be interpreted as follows. At high dilution, less abundant compounds fall below the detection limit. For instance, in the macrocrystalline sample at 2 wt.%, nine fewer compounds were detected compared to the 10 wt.% sample (two in the light paraffin range and seven in the heavy paraffin range), leading to an increase in the relative error. Conversely, increasing the stock concentration produces two competing effects: It expands the detectable C# range but also promotes paraffin aggregation, which compromises HTGC quantification. For example, in the macrocrystalline sample at 20 wt.%, three additional heavy compounds were detected relative to the 10 wt.% sample; however, the relative error was considerably lower for the 10 wt.% sample, highlighting a trade‐off between detection range and aggregation effects. These findings indicate that concentrating the sample is beneficial for qualitative analysis, as it broadens the detectable paraffin range, but may introduce significant errors in quantitative analysis. Therefore, for quantitative purposes, an optimal balance between dilution and concentration must be carefully selected to minimize analytical error.

Across all concentration levels, the pure microcrystalline system, mainly composed of heavier paraffins, exhibited consistently high relative errors, exceeding 40% in all cases. This behavior can again be attributed to poor sample homogeneity caused by paraffin aggregation, even under diluted conditions. Consequently, when analyzing pure microcrystalline samples with the present HTGC setup, the results should be interpreted primarily from a qualitative perspective, with substantial limitations for reliable quantification.

Interestingly, the relative error of the mixed system was lower than that of the pure macrocrystalline system for the three most diluted samples, despite half of its composition consisting of microcrystalline paraffins, which, when analyzed alone, exhibited large errors. This observation further supports the conclusion drawn in the previous section that heavy paraffins enhance the effective solubility of light paraffins, thereby improving sample homogeneity. In contrast, for the two most concentrated mixed samples, the relative errors exceeded those of the pure macrocrystalline system. This suggests that, within this concentration range (≈2 × 10−2 wt.%), which is higher than that studied in the previous section, the aggregation driven by the increased fraction of microcrystalline paraffins outweighs the solubility enhancement of light paraffins. In addition, the high concentration of heavy paraffins in the sample may hinder the effective volatilization of analytes, further contributing to the increase in relative error.

**FIGURE 6:**
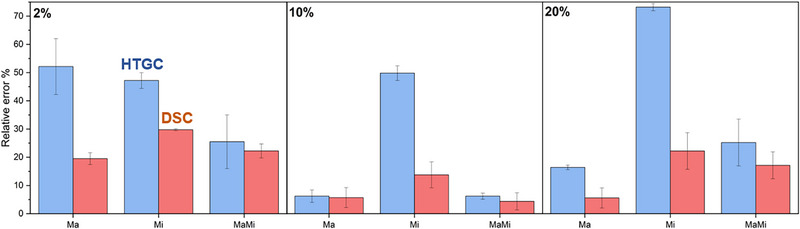
Comparison between the relative errors obtained in the analysis through HTGC (blue) or DSC (red). “Ma” relates to the pure macrocrystalline sample. “Mi” relates to the pure microcrystalline sample. “MaMi” relates to the 1:1 mixed sample. The total paraffin concentrations of the samples are showed in the top left side of each graphic. DSC, differential scanning calorimetry; HTGC, high‐temperature gas chromatography.

To further support these interpretations, the HTGC results were compared with those obtained using an established DSC method for determining paraffin wax concentrations in solution [24]. The comparative results are presented in Figure 6.

Two main observations can be drawn from Figure 6. First, when comparing samples with identical paraffin composition but different concentrations, the relative errors obtained from the DSC analysis also exhibit the approximately parabolic trend observed in the HTGC results. This behavior indicates that, as both methodologies rely on similar sampling procedures based on the fractionation of paraffin solutions, errors arising from insufficient sample homogenization affect both techniques. Second, the relative errors associated with the DSC methodology are lower than those obtained by HTGC. This difference suggests the presence of an additional significant source of error in HTGC analysis, most likely related to the incomplete volatilization of heavy paraffins.

On the basis of these results, three key considerations can be highlighted: (1) The enhancement of light paraffin solubility in the presence of heavy paraffins, previously reported using other experimental techniques [27, 28], directly impacts chromatographic performance by improving sample homogenization. (2) For quantitative analysis, a careful balance between dilution and concentration is required, as both introduce competing effects: Excessive dilution may cause the loss of less abundant compounds below the detection limit, whereas excessive concentration promotes paraffin aggregation, thereby reducing analytical accuracy. For qualitative analysis, concentrating the sample is recommended to maximize the detectable carbon number range. (3) Two main sources of error affect HTGC analysis of paraffins in methodologies based on ASTM D5442: insufficient homogeneity of the paraffin solution and incomplete volatilization of all analytes, particularly heavy paraffins. It is worth noting that the considerations discussed here are also applicable to crude oil characterization, which typically involves systems with high C# polydispersity.

### 3.3 Effect of Paraffin Molecular Structure

Considering the importance of accounting for nonlinear paraffinic compounds to better understand the physicochemical properties of waxy systems and improve predictions of the wax deposition, this section investigates the limitations that should be considered when using a one‐dimensional HTGC–FID experimental setup for the characterization and quantification of these compounds. Although some studies in the literature provide a detailed characterization of all hydrocarbon groups in crude oil samples [8, 11], these were conducted using comprehensive two‐dimensional GC and mass spectrometry, which differs from the scope of the present work, focused on one‐dimensional HTGC–FID.

The FID detector alone cannot determine the molecular structure of the detected hydrocarbons, as it relies on external standards to assign a given compound to a chromatographic peak. As analytical standards are not available for all the numerous hydrocarbons present in a crude oil sample, the objective of this study is to evaluate whether systematic patterns can be extracted from HTGC–FID data to enable the characterization and quantification of nonlinear paraffins.

**FIGURE 7:**
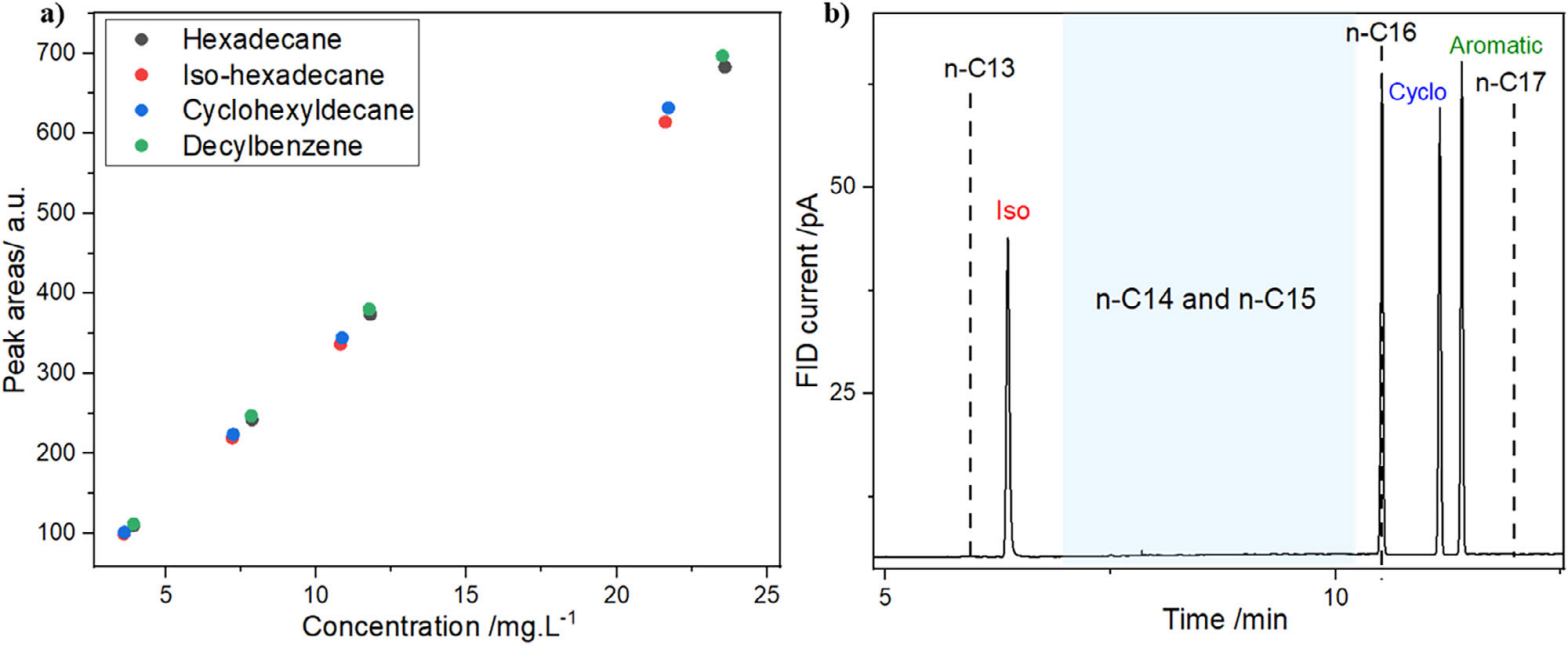
Chromatographic data of distinct molecular morphologies of C16. (a) Calibration curves for quantitative analysis. (b) Chromatogram containing the four distinct molecules of C16. FID, flame ionization detection.

Figure 7a presents the calibration curves obtained for the different molecular structures of C16 paraffins (linear, branched, naphthenic, and aromatic). The slopes of the fitted calibration curves are very similar for all four isomers. This behavior is expected, as the FID response is primarily proportional to the carbon number of the analyte and is largely independent of its molecular structure. On the basis of these results, in the absence of a specific analytical standard for a given isomer, the calibration curve of another isomer may be used as an approximation for its quantification, within the uncertainty inherent to the method.

The possibility of establishing a systematic pattern that would allow the chromatographic methodology to assign a peak to a probable molecular structure in the absence of external analytical standards was then evaluated. In Figure 7b, the naphthenic and aromatic C16 compounds exhibit slightly longer retention times than their linear C16 counterpart, while still eluting before linear C17. In contrast, the branched C16 compound shows a markedly shorter retention time compared to the other C16 isomers, barely exceeding that of linear C13. This behavior strongly limits the feasibility of establishing a general retention time‐based trend for peak assignment of nonlinear isomers. Since the retention time of branched C16 overlaps with the retention time window of naphthenic and aromatic C13, a similar trend is expected across other carbon numbers, making it unreliable to distinguish molecular structures based solely on retention time patterns.

These results provide important insights by showing that it is not accurate to assume that all components between *n*‐C15 and *n*‐C16 correspond to C16 isomers, a simplification commonly adopted in chromatographic characterizations associated with pressure–volume–temperature (PVT) analysis, an important characterization of crude oils that provides its physicochemical properties in reservoir conditions [29].

Additional observations can further weaken the possibility of establishing a clear trend for assigning chromatographic peaks to nonlinear compounds. First, only a single branched compound related to C16, namely, 2,2,4,4,6,8,8‐heptamethylnonane, was evaluated in this study. However, numerous other branched isomers exist for C16, which would further enhance the complexity of the retention time profile. Second, for higher C#, additional hydrocarbon groups such as polyaromatics and polycyclic naphthenes must be considered. The presence of these compounds would further increase the complexity of the retention time distribution within each C#, making it even more challenging to establish a retention time pattern that does not overlap with compounds from other C#.

In this highly complex scenario with extensive peak overlapping, comprehensive two‐dimensional gas chromatography (HTGC×GC) emerges as a valuable alternative to improve chromatographic resolution as it enables more reliable characterization of nonlinear paraffinic compounds.

## 4 Conclusion

In the literature, the conventional sample preparation methodology for the characterization and quantification of paraffins from diverse matrix consists in solubilizing the paraffins in an organic solvent followed by injection in the HTGC–FID. Achieving effective solubilization of paraffinic compounds remains challenging even at temperatures above the WAT, resulting in solutions that are nonhomogeneous at the colloidal scale and, therefore, lack analytical representativeness. Consequently, this sample preparation procedure introduces significant uncertainties and systematic errors into the analysis.

A solvent‐based sample preparation methodology is prescribed by ASTM D5442. Within this context, the present study systematically investigated the capabilities and limitations of one‐dimensional HTGC–FID for the quantification of paraffinic compounds across distinct paraffin systems. In addition, the potential driving factors behind the observed analytical errors were examined by comparing the HTGC results with those obtained using an established DSC method for determining paraffin content in solution. The results demonstrate that two primary sources of error must be considered when quantifying paraffins by HTGC: the lack of effective homogeneity in the paraffin solution and the incomplete volatilization of analytes, particularly in systems containing heavy paraffins.

The results further highlight a clear relationship between molecular weight and chromatographic quantification error. Both light and heavy paraffins exhibit aggregation in solution, which adversely affects HTGC performance.

However, aggregation is substantially more pronounced for heavy paraffins. For light paraffins, the HTGC quantification error increases with concentration, whereas the opposite trend is observed for heavy paraffins. Overall, the quantification error associated with heavy paraffins is considerably higher than that for light paraffins, underscoring the intrinsic difficulty of accurately characterizing high carbon number species by one‐dimensional HTGC–FID.

With respect to the total paraffin content in the sample, over‐dilution increases analytical error because less concentrated components fall below the detection limit. Conversely, over‐concentration also increases error by enhancing paraffin aggregation, thereby compromising solution homogeneity and worsening sampling representativeness. Therefore, when quantitative analysis is required, the opposing effects of dilution and concentration must be carefully balanced to identify an optimal working concentration, which is system‐dependent and varies with paraffinic composition. When a qualitative analysis is the primary objective, concentrating the sample is recommended. Under the experimental conditions employed in this work, concentrations on the order of ≈2 × 10−1 wt.% maximize the detectable carbon number range.

Previous reports using other experimental techniques have demonstrated that heavy paraffins enhance the solubility of light paraffins. Since an important source of error in HTGC quantification arises from poor sampling due to insufficient homogeneity, this solubility effect plays a crucial role in improving analytical accuracy. In this work, this phenomenon was examined in two distinct scenarios: a model system with C20 and C40 and mixed systems composed of macrocrystalline and microcrystalline paraffins. In both cases, the presence of heavy paraffins improved the quantification accuracy of light paraffins in HTGC analysis.

Finally, no consistent retention time patterns were identified that would allow reliable discrimination of paraffin molecular structures in the absence of external analytical standards. Given the importance of nonlinear paraffins in defining the physicochemical properties of wax deposits, their accurate characterization and quantification require more advanced analytical approaches, such as coupling HTGC with mass spectrometry or comprehensive two‐dimensional gas chromatography.

## Author Contributions

**Fernando B. Okasaki**: conceptualization, writing – original draft, investigation, visualization. **Ivanei F. Pinheiro**: conceptualization, writing – reviewing and editing. **Letícia Bizarre**: conceptualization, writing – reviewing and editing. **Vanessa C. B. Guersoni**: conceptualization, supervision, funding acquisition, writing – reviewing and editing.

## Supporting information




**Supporting File**: jssc70382‐sup‐0001‐SuppMat.docx.

## Data Availability

The data that support the findings of this study are available from the corresponding author upon reasonable request.
