# Supplementary material for: Evaluating the Limitations of One‐Dimensional High‐Temperature Gas Chromatography ‐ FID for Wax Solution Analysis: A Systematic Study
Source: J Sep Sci. 2026 Mar 4;49(3):e70382. doi: 10.1002/jssc.70382 (PMC12961180; doi:10.1002/jssc.70382)
Supplement: Supplementary file 1 — Supporting File: jssc70382‐sup‐0001‐SuppMat.docx. [file JSSC-49-e70382-s001.docx]

**Supplementary Information**

**For**

**Evaluating the limitations of HTGC-FID for wax solution analysis: a systematic study.**

Fernando B. Okasaki^1,2,*^, Ivanei F. Pinheiro^2^, Letícia Bizarre^2^, Vanessa C. B. Guersoni^2^

1 Faculdade de Engenharia Mecânica, Universidade Estadual de Campinas – UNICAMP. Campinas, SP, Brazil.

^2^ Centro de Estudos de Energia e Petróleo – CEPETRO, Universidade Estadual de Campinas – UNICAMP. Campinas, SP, Brazil.

*Corresponding author: Fernando Bonin Okasaki, fernandobonin96@gmail.com

**Parameter optimization**

A crude oil solution in cyclohexane with concentration of 1 mg mL^−1^ was used in all experiments of this section.


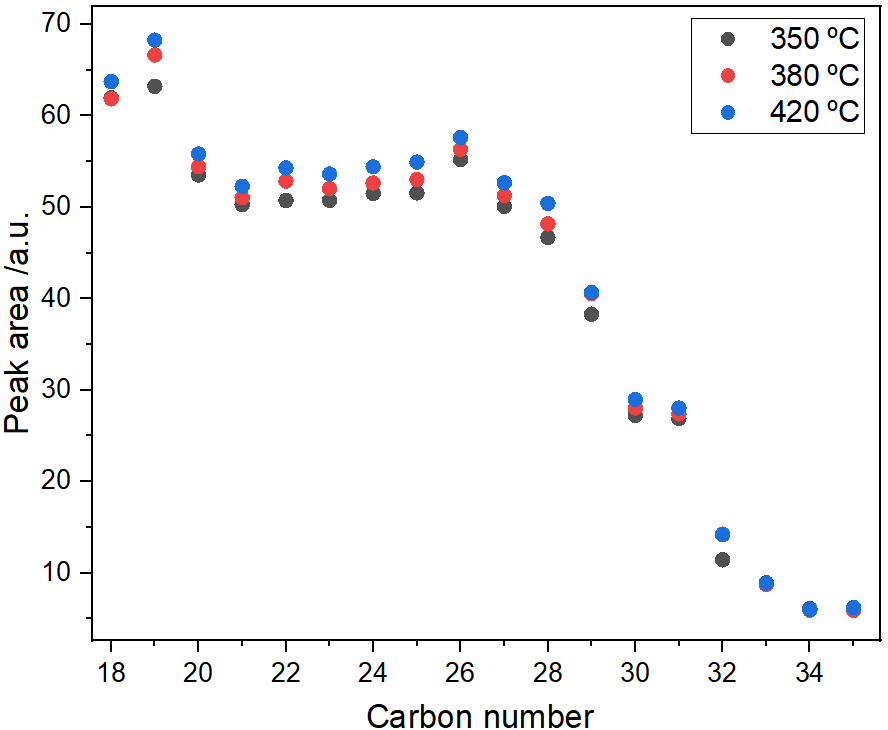


**Figure S1.** Peak areas obtained for different carbon number compounds at different FID temperatures (indicated in the figure).

The marginal increase obtained at 420 ºC does not justify the decrease in equipment health as the maximum column operating temperature recommended by the manufacturer is 400 ºC


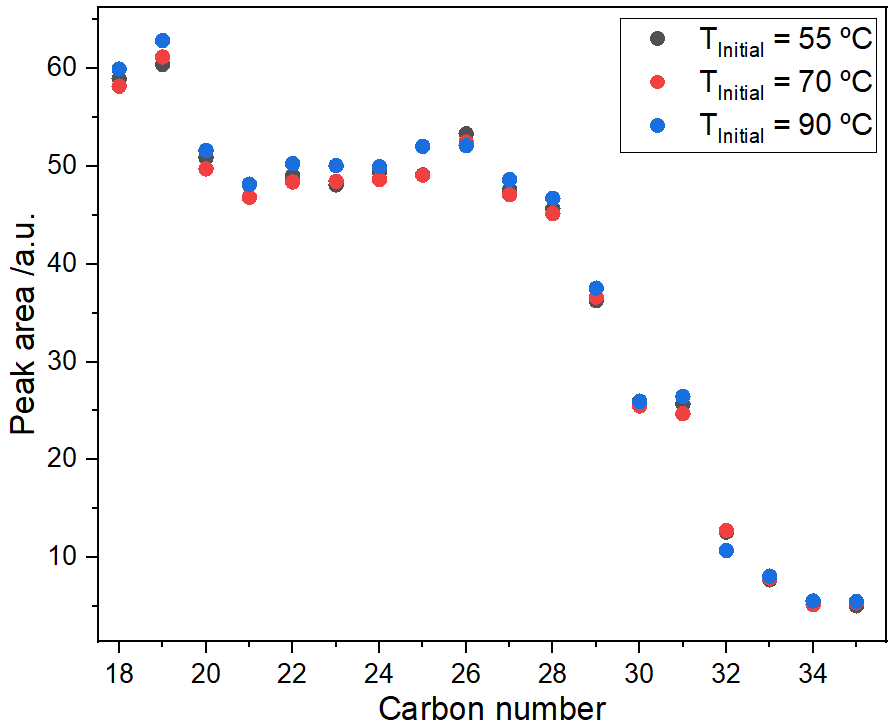


**Figure S2.** Peak areas obtained for different carbon number compounds at different initial column temperatures (indicated in the figure).

Increasing the initial column temperature does not significantly alters the C# distribution profile nor its values. The initial temperature of 90ºC was selected in order to reduce the duration of the experimental procedure.


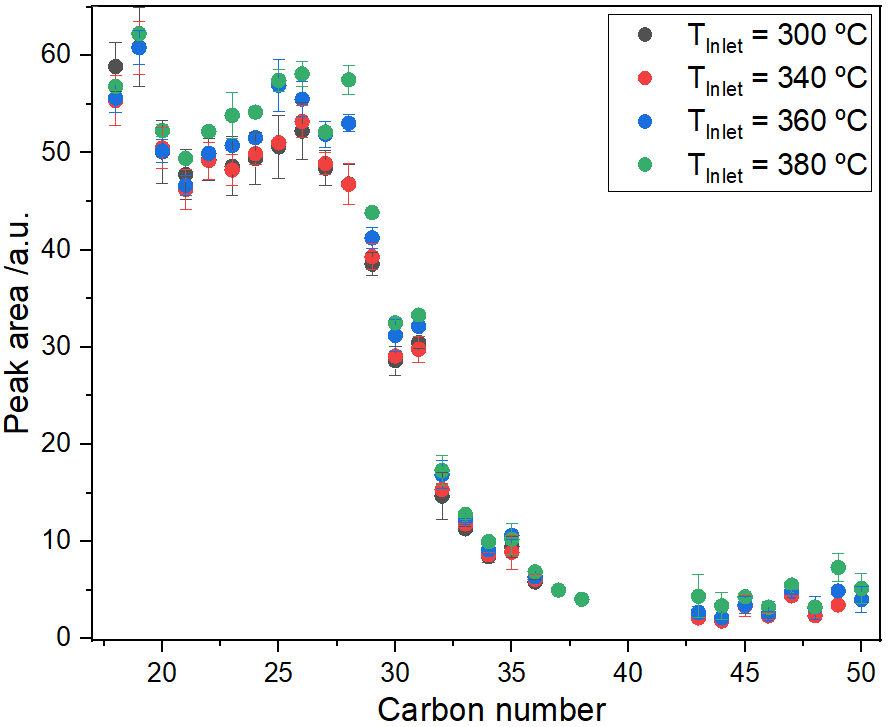


**Figure S3.** Peak areas obtained for different carbon number compounds at different inlet temperatures (indicated in the figure).

If an insufficient inlet temperature is employed, less volatile compounds may not be efficiently transferred into the column, leading to incomplete sample introduction. Increasing the inlet temperature results in a substantial improvement in both the detectable C# range and the peak areas. The final inlet temperature was set to 380 °C, remaining below the column’s maximum operating temperature of 400 °C.


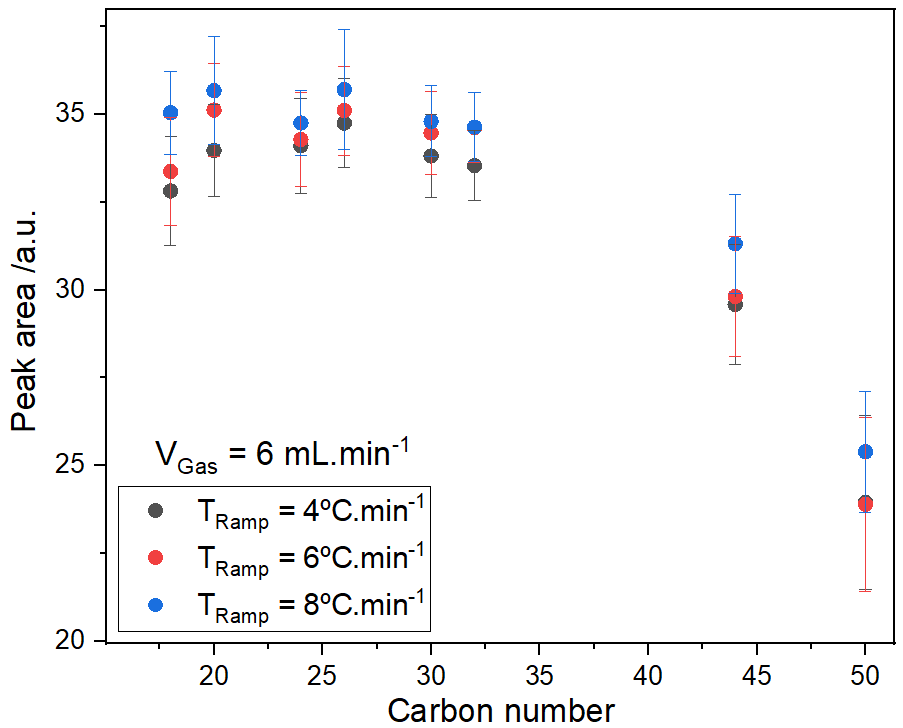


**Figure S4.** Peak areas obtained for different carbon number compounds at different temperature ramps (indicated in the figure).

The temperature ramp does not considerably impact in the peak areas and detectable C# range. The condition of 8 ºC.min^-1^ was chosen as it promotes a satisfactory resolution between the peaks.

**Supplementary material**


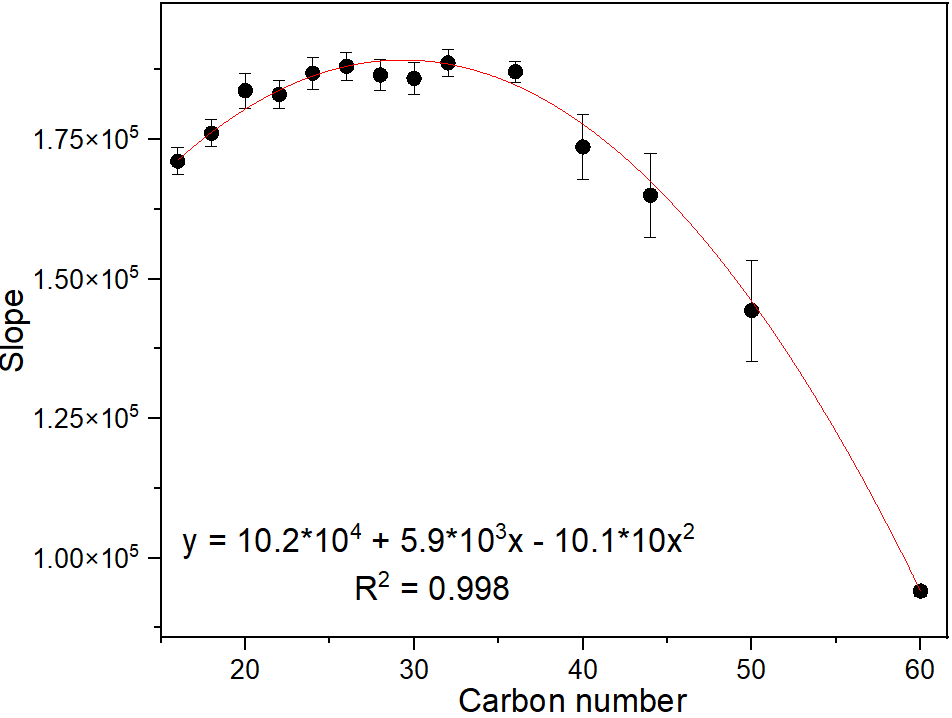


**Figure S5.** Experimental slopes obtained through the calibration curves (black dots) adjusted with a polynomial fit (red line). The equation of the polynomial fit is shown in the inlet.


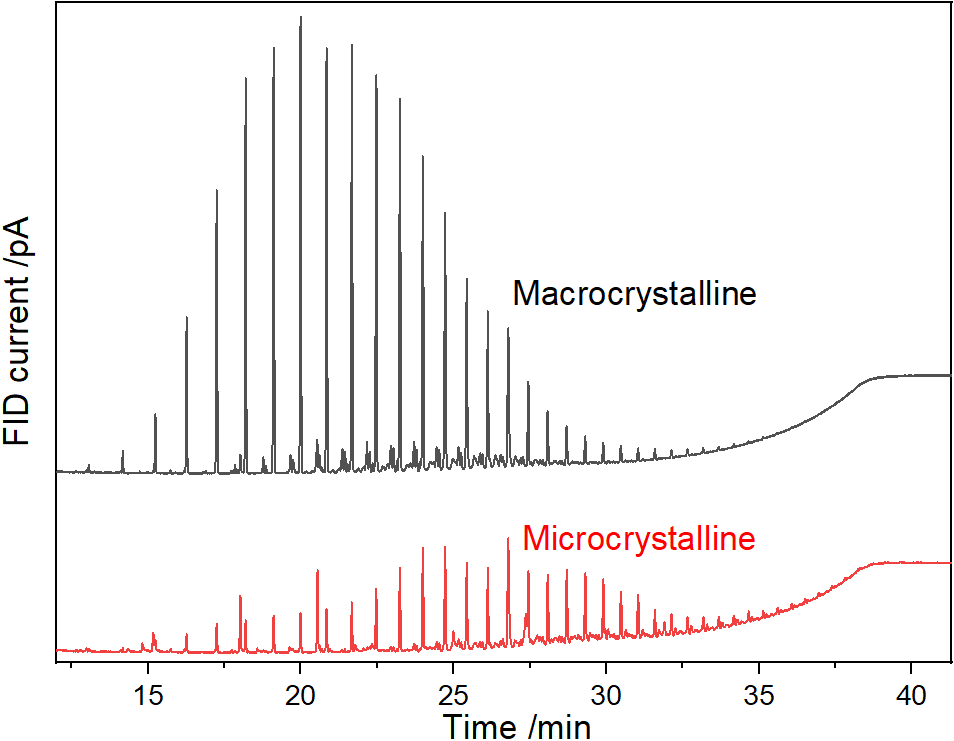


**Figure S6.** Chromatograms obtained for 10 wt.% solutions of macrocrystalline wax (black) and microcrystalline wax (red).

**Turbidity measurements**

Turbidity measurements were carried out in an Anton-Paar Litesizer DLS 500®, equipped with a laser diode of 40 mW and wavelength of 658 nm. A quartz cuvette was used as the sample holder. Temperature was maintained at 25 °C. Prior to the measurement, the sample was submitted to an equilibration step of 10 s in order to guarantee the thermal equilibrium in the system. The turbidity was then measured for 30 s. Three samples were measured: pure cyclohexane, C16 at 5.10^-3^ wt.\% and C40 at 5.10^-3^ wt.\%. All measurements were conducted in triplicate.

| **Sample** | **Transmittance %** |
| --- | --- |
| Cyclohexane | 91.40 ± 0.01 |
| C16 | 91.10 ± 0.04 |
| C40 | 91.41 ± 0.02 |
